# Histological Features of the Olive Seed and Presence of 7S-Type Seed Storage Proteins as Hallmarks of the Olive Fruit Development

**DOI:** 10.3389/fpls.2018.01481

**Published:** 2018-10-12

**Authors:** Adoración Zafra, Mohammed M’rani-Alaoui, Elena Lima, Jose Carlos Jimenez-Lopez, Juan de Dios Alché

**Affiliations:** ^1^Plant Reproductive Biology Laboratory, Department of Biochemistry, Cell and Molecular Biology of Plants, Estación Experimental del Zaidín, Granada, Spain; ^2^Département de Biologie, Université Abdelmalek Essaadi, Tétouan, Morocco

**Keywords:** β-conglutins, cotyledon, development, endosperm, olive, radicle, seed, seed storage proteins

## Abstract

The production of olive oil is an important economic engine in the Mediterranean area. Nowadays, olive oil is obtained mainly by mechanical processes, by using the whole fruit as the primary raw material. Although the mesocarp is the main source of lipids contributing to olive oil formation, the seed also contributes to the olive oil composition and attributes. The olive seed is also becoming an interesting emerging material itself when obtained after alternative processing of the olive fruit. Such seed is used for the production of differential oil and a unique flour among other bioactive products, with increasing uses and applications in cosmetics, nutrition, and health. However, olive seed histology has been poorly studied to date. A complete description of its anatomy is described for the first time in the present study by using the ‘Picual’ cultivar as a model to study the development of the different tissues of the olive seed from 60 to 210 days after anthesis. A deep analysis of the seed coats, endosperm storage tissue and the embryo during their development has been performed. Moreover, a panel of other olive cultivars has been used to compare the weight contribution of the different tissues to the seed, seed weight variability and the number of seeds per fruit. In addition to the histological features, accumulation of seed storage proteins of the 7S-type (β-conglutins) in the seed tissues has been assessed by both biochemical and immunocytochemical methods. These hallmarks will help to settle the basis for future studies related to the location of different metabolites along the olive seed and mesocarp development, and therefore helping to assess the appropriate ripening stage for different commercial and industrial purposes.

## Introduction

Alimentary industries based in the preparation of table olives and olive oil are of paramount importance for the economy of Mediterranean countries and some areas of America and Australia. The very well valued Extra Virgin Olive Oil (EVOO) is produced exclusively by mechanical processes where the whole fruit is used. Other olive oil qualities [Virgin Olive Oil (VOO) and Ordinary Virgin Oil (OVO)] are also produced mainly from whole fruits as the primary raw material. Thus, the obtained juices also contain components from the seed, which contribute to olive oil aroma and other potential properties like peroxidase activity (Luaces et al., 2003, 2007). The olive seed itself is also becoming an interesting material with multiple uses beginning to emerge (Rodríguez et al., 2008; Matos et al., 2010; Pattara et al., 2010; Naghmouchi et al., 2015). Morphological characteristics of the olive pit have been used as descriptors of pomological interest for varietal characterization in the olive tree (Barranco and Rallo, 1984). However, differences between the seed have not been reported in detail to our knowledge.

The histology of the different tissues of the seeds have been described in a variety of species other than olive (*Olea europaea* L.). In these studies, the structure of the seed coat was one of the most widely topics described in the literature. Early in the thirties, a deep study on the almond seed surface was performed aimed to easily distinguishing the different varieties of almonds, hence helping identifying misrepresentation or adulteration (Pease, 1930). Examination of *Arabidopsis* seed coat development showed major morphological changes associated with the transition of the integuments into the mature seed coat (Beeckman et al., 2000). Analysis of the seed coat histological distribution has also been performed in *Cucurbita pepo* L. to examine mutations concerning the lignification of the testa (Zraidi et al., 2003). Similarly, the seed coat of *Chenopodium quinoa* was histologically studied aimed to assess and improve quality of the seeds for human and animal consumption (Raamsdonk et al., 2010). The seed coat form of other species such as *Passiflora ligularis* Juss or *Strychnos potatorum* L. has also been analyzed (Cárdenas-Hernández et al., 2011; Mishra and Vijayakumar, 2015). Regarding endosperm anatomy, a new approach in the disclosure of the history of flowering plants has been provided after comparison of the patterns of endosperm development as well as analysis of phylogenetic and ontogenic evolution of this tissue using several basal flowering plants (Floyd and Friedman, 2000). The histology of the seeds from plants like *Vitis vinifera* L., *Paronychia*, *Theobroma cacao* L., *Annona squamosa* L., and *Medicago truncatula* has been described (Cadot et al., 2006; Kaplan et al., 2009; Rangel-Fajardo et al., 2012; Martínez et al., 2013; Verdier et al., 2013). The structure and storage content of *Arabidopsis* and *Cuphea glutinosa* endosperms has also been scrutinized (Li et al., 2006; Di Santo et al., 2012). Finally, the anatomy of the cotyledons has been particularly studied in *Theobroma cacao* L. and *Eurycoma longifolia* seeds (Elwers et al., 2010; Danial et al., 2011), where descriptions of the pattern of distribution of the polyphenolic compounds and the development of the vascular system have been provided. By means of non-destructive techniques, the structure of whole seeds has been also examined. As result, valuable information about the transport system for gas exchange in embryos of the *Arabidopsis* seed has been provided (Cloetens et al., 2006). Similarly, a 3D reconstruction of the compartments present in the maize seed have been performed (embryo, endosperm, nucellus, and pericarp) from 7 to 21 days after pollination (Rousseau et al., 2015).

The number of studies focused on the olive seed histology is still reduced. Initial studies dealt with the description of morphological, histological, and ultrastructural changes in the olive pistil during flowering (Suárez et al., 2012), and the localization of seed storage proteins (SSPs) in the olive seed. SSPs are synthesized in abundance in the developing seeds and are accumulated primarily in the protein storage vacuoles (PSVs) of terminally differentiated cells of embryo and endosperm (Herman and Larkins, 1999). Previous reports indicate that mature olive seeds contains very similar subcellular structure in both the embryo and endosperm tissues, essentially with electro-dense protein bodies (PBs) surrounded by lipid bodies with diameters ranging from 0.5–2.0/μm (Ross et al., 1993; Zienkiewicz et al., 2014). The endosperm and the cotyledon are considered storage tissues, where members of the 11S protein family are the most abundant from the total of seed proteins (Alché et al., 2006). However, asynchrony exists in the formation of PBs between both tissues (Jiménez-López et al., 2015). The analysis of the protein synthesis along the seed formation has determined three periods: (I) a period of early synthesis (before 105 days after anthesis, DAA), (II) a rapid and massive period of synthesis (105–130 DAA), and (III) a period characterized by slow synthesis (from 130 DAA until full ripening) (Wang et al., 2001).

Authors have also fixed their attention to describe the intracellular events occurring during the first hours of the *in vitro* germination process (Zienkiewicz et al., 2011; Jiménez-López and Hernández-Soriano, 2013), drawing their attention particularly to PBs. Zienkiewicz et al. (2011) also revealed that the cellular organization of the olive leaf is achieved after 26 days of germination.

β-Conglutins, vicilins of 7S globulins are also major SSPs in different plants, particularly legumes. Among them, they have been particularly studied in Lupinus species (Jimenez-Lopez et al., 2015, 2016, 2018; Lima-Cabello et al., 2017a,b, 2018). They belong to the Cupin superfamily, and mainly associate (as storage protein function) with plant physiological processes through the supply of amino acids during seedling germination (Monteiro et al., 2010). Primary evidence of the presence of β-conglutins in the olive arise from transcriptomic analyses, as the presence of 7S globulins transcript sequences have been detected in the olive seed (unpublished results). However, direct evidence of the presence and distribution of β-conglutins in the olive seed has not been provided to date.

In spite of these pioneer studies, an overall histological description of the olive seed is yet missing to date. Here we perform a report of the different tissues of the olive seed throughout its development and we use a new molecular tool: the 7S SSPs (β-conglutins) recently described in the olive seed, and a specific antibody developed to evidence the presence of these proteins and their changes as markers along tissue development. The results shown here may serve as a hallmark for analyzing seed (and fruit) maturity and to monitor the presence of these proteins in future biotechnological and alimentary uses due to their increasing interest. Finally, cell localization of these proteins is also reported.

## Materials and Methods

### Plant Material

Seeds used for microscopy analysis were collected from olive trees (*Olea europaea* L. cv. ‘Picual’) cultivated at the Estación Experimental del Zaidín (Granada). Four stages were considered: (0) small developing fruit, (I) green fruit, (II) fruit at veraison, and (III) mature fruit. The collection took place 60, 105, 130, and 210 DAA, respectively. Seeds from different cultivars were kindly provided by the Protected Certificate of Origin “Poniente de Granada.” The cultivars studied were ‘Ombliguillo,’ ‘Llorón,’ ’3,’ ‘Lechín,’ ‘Hojiblanca,’ ‘Picual,’ ‘Lucio,’ ‘Alameño,’ ‘Nevadillo,’ ‘Loaime,’ ‘Azul,’ and ‘Gordal de Alhama.’ Twenty fruits per cultivar were dissected by using a knife, a de-stoning commercial device, and a scalpel to dissect the pulp (mesocarp + epicarp), stones and the seed tissues respectively. Weight measurements were performed individually using 20 samples of the complete mature fruit (210 DAA) and each one of the dissected tissues [mesocarp + epicarp, whole endocarp (stone), testa, endosperm, and embryo]. The number of seeds obtained from each fruit was also counted.

### Preparation of Samples for Microscopy

**FIGURE 1:**
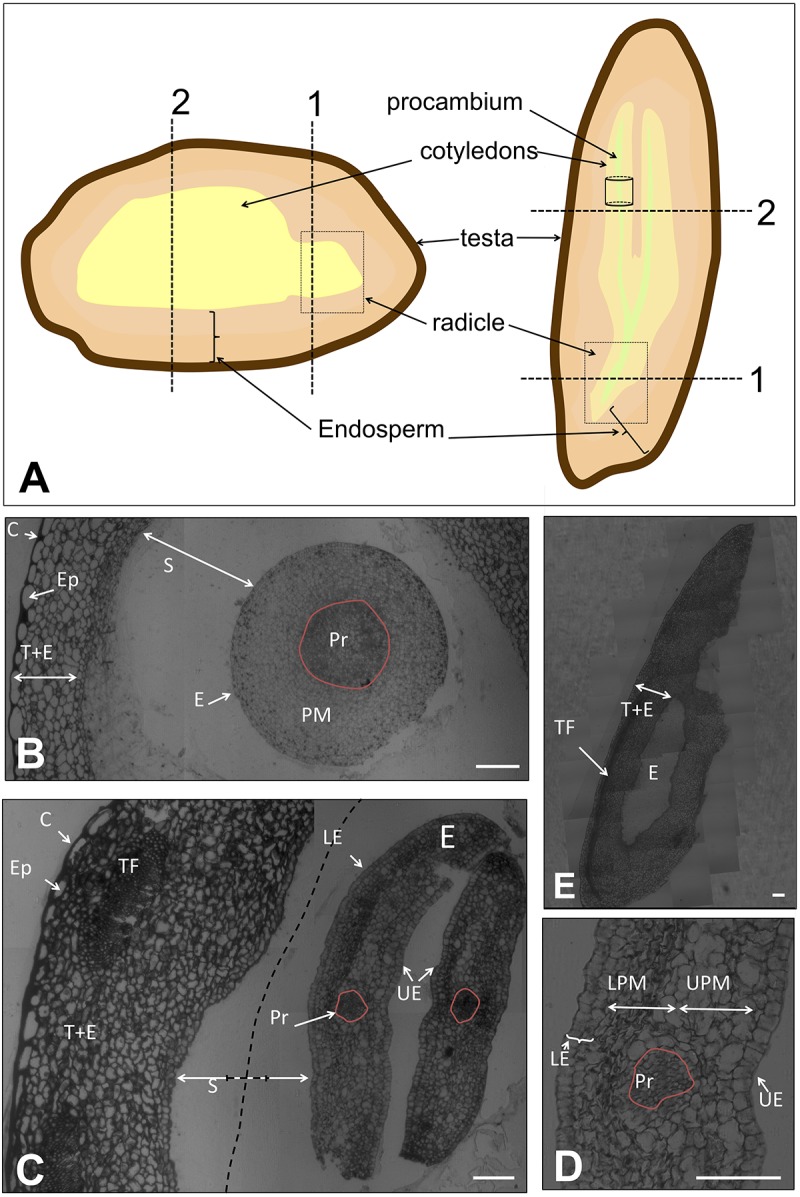
**(A)** Representation of the tissues of the olive seed in longitudinal- versus cross-sections. Dotted lines/boxes show the orientation of the samples for microscopy analysis. **(B)** Cross section of the complete seed at the radicle site (pointed out with “1” in **A**) at 60 DAA. **(C)** Cross section of the complete seed at the cotyledon site (pointed out with “2” in **A**) at 60 DAA. Dotted lines indicate the shortening of the space between endosperm-embryo. **(D)** Detail of the cotyledon observed in panel **(C)**. **(E)** Longitudinal section of the complete seed at 60 DAA. Pr, procambium; PM, premesophyll; E, embryo; S, space between endosperm-embryo; C, cuticle; Ep, epidermis; T+E, testa and endosperm; TF, transversal fibers; LE, lower epidermis; UE, upper epidermis; UPM, upper premesophyll cells; LPM, lower premesophyll cells. Scale bars: 100 μm.

Seeds from olive fruits at four developmental stages were collected. The mesocarps + epicarps (pulp) and the endocarps (stones) were removed with a knife and a de-stoning device, respectively. At the stage 0, the complete seed was used. In the rest of the stages the obtained seeds were carefully dissected into two parts: on the one hand the coat and the endosperm were treated together, on the other hand the embryo was carefully excised. Once the embryo was obtained, the apical part (radicle) and the middle part (cotyledons) were treated separately (**Figure 1**). The plant materials were fixed with 4% (w/v) paraformaldehyde and 0.2% (v/v) glutaraldehyde in 0.1 M cacodylate buffer (pH 7.2) for 2 h at 4°C with points of vacuum treatment to improve penetration of the fixative. Samples were dehydrated in ethanol series and embedded in Unicryl resin at −20°C using ultraviolet light. Semithin sections were obtained with a Reichert-Jung Ultracut E microtome using a glass knife. Sections were placed on Biobond-coated slides and used for cytochemical staining.

### Histological Study

For histological observations, sections were stained with a mixture of basic dyes [0.05% (w/v) methylene blue and 0.05% (w/v) toluidine blue] aimed to stain the carboxyl groups of proteins, which reveal the presence of such components. Most non-stained structures correspond to lipids. Stained samples were observed in a LM Zeiss Axioplan (Carl Zeiss, Oberkochen, Germany). Photomicrographs were obtained with a ProgRes MF Cool Digital Camera, by using the ProgRes CapturePro 2.6 software (Jenoptik, LaserOptic System).

### Development of an Anti-β-conglutin Antibody

Olive transcriptomic information together with sequence information of β-conglutins from different species was used to define potential cross-reactive epitopes of these proteins present in these species (Jimenez-Lopez et al., 2015). The peptide RLENLQNYRIVEFQS was selected as a cross-reactive component on this basis and was synthesized and used to immunize rabbits by Agrisera (Sweden) (Prod. No. AS15 2892). The resulting sera were affinity-purified with the synthetic peptide, and their specificity assessed by Western blotting and ELISA (not shown).

### Protein Extraction and Western Blotting Analysis

Plant material (as described) was used to prepare protein extracts by grinding with liquid nitrogen. Proteins were extracted with 40 mM Tris–HCl pH: 7.0, 2% Triton X-100, 60 mM DTT and 10 μl/sample of protease inhibitor cocktail (Sigma). Samples were denatured with Laemmli sample buffer at 95°C for 5 min and separated on 4–20% TGX precast SDS-PAGE mini-gels (Bio-Rad). Protein profiles were determined by means of Stain-free technology using a Gel Doc^TM^ EZ System (Bio-Rad), and normalized for total protein (30 μg/lane). Gels were blotted to supported nitrocellulose using a Trans-Blot Turbo (Bio-Rad) semi-dry device and blocked with 5% skimmed milk in TBS plus 0.05% Tween-20 for 1 h at room temperature (RT) with agitation. Blot was incubated in the anti-β-conglutin primary antibody at a dilution of 1:1000 for 8 h at 4°C with agitation in TBS-T plus 5% skimmed milk. The antibody solution was decanted and the blot was rinsed briefly twice, then washed once for 15 min and 3 times for 5 min in TBS-T at RT with agitation. Blot was incubated in secondary antibody [anti-rabbit IgG horseradish peroxidase conjugated, from Sigma (A-0545)] diluted to 1:2000 in for 1h at RT with agitation. The blot was washed as above and developed for 3 min with Clarity Western ECL substrate (Bio-Rad). Exposure time was 6–12 min in a C-Digit scanner (LI-COR Biotechnology, United States). The intensity of the reacting bands and their approximate Mw was determined with the Image Studio^TM^ software (LI-COR Biotechnology, United States) as the average ± SD of three experiments.

### TEM Immunolocalization of Olive 7S SSPs (β-Conglutins)

Ultrathin sections (70 nm) were obtained using a Reichert-Jung ultramicrotome and picked up using 200 mesh nickel grids coated with formvar. The grids were then sequentially treated with a blocking solution [5% (w/v) bovine serum albumin, 0.1% (v/v) Tween 20 in phosphate-buffered saline], a diluted (1:100) solution of the anti-7S antiserum in blocking solution, a 1:1000 solution of the secondary antibody (goat anti-rabbit IgG: 30 nm gold, BB International), and finally contrasted using a 5% (w/v) uranyl acetate alternative solution (Ted Pella Inc., CA, United States) and observed in a JEM-1011 (Jeol) transmission electron microscope (TEM). Negative control sections were treated as above but using preimmune serum instead of the anti-conglutin antiserum. Morphometric measurements were performed using the UTHSCSA ImageTool (version 3.00 for Windows) software.

### Statistical Analysis

The Kolmogorov–Smirnov test was used to test the normality of all weight parameters. The Pearson test was performed aimed to determine whether whole fruit and mesocarp weight were correlated. For Western blotting and immunocytochemical analysis, values expressed as mean ± SEM of individual experiments were assessed for statistical significance of the data by analysis of variance followed by Dunnett’s analysis. *P*-values ≤0.001 were considered statistically significant. All analyses were performed using IBM SPSS statistics v.24 software.

## Results

### Olive Seed Anatomy at Early Stages of Fruit Development

The complete seed was processed 60 DAA to visualize general structure at a very early stage of development. At this moment, dissection of the seed into its tissues was not achievable without tissue damage due to the small size of the seed and the high compaction of the tissues. In **Figure 1A**, a schematic draw of the different tissues of the olive seed is displayed, as well as the positions selected for longitudinal- and cross-sections performed in this study.

A cross section of the complete seed at the radicle level showed that the testa and the endosperm tissues were immature, without appreciable differentiation among these two tissues (**Figure 1B**). The cells appeared unstained, indicating no clear accumulation of storage material neither in the endosperm nor in the embryo, as previously described (Jiménez-López et al., 2015).

No presence of the aleurone layer was detected. However, the presence of the cuticle and the pro-epidermal layer cells from the testa was visible. The cuticle was evidenced by an intense staining with methylene blue at the outermost site. The pro-epidermal layer of cells was placed under the cuticle, composed of long-shaped cells. Regarding the embryo, isodiametric cells were observed with slight differences among them. In the center of the embryo, the cells appeared intensely stained, this central structure corresponding to the precambium. The premesophyll cells were located surrounding those of the precambium. The pre-dermal cells appeared in the outer part, characterized by the presence of notorious nuclei. The embryo and the endosperm were separated by an ample space that remained unstained (**Figure 1B**). Similarly, a cross section of the embryo at the cotyledon level showed that the testa and the endosperm appeared undifferentiated. However, in this area, the presence of transversal fibers was patent. The thickness of the precursor of the testa and endosperm at the cotyledon level was approximately twofold that at the radicle level. The width increment was due to both, the presence of transversal fibers, and the rise in the number of cells (**Figure 1C**). The embryo cross-section at the level of the cotyledon showed cells with a quite marked differentiation (**Figure 1E**). Four types of cells were observed: those forming the procambium, the upper epidermis, the lower epidermis, and the premesophyll (**Figures 1C,E**). It was observed that the upper and lower epidermis contained one and two layers of cells, respectively, in both cases with a cubic shape. On the other hand, the premesophyll contained non-stained cells with variable shape and size. The presence of the procambium cells was evidenced as a group of small and densely packed cells among the premesophyll. A longitudinal section of the complete seed showed the position of the embryo within the seed as well as the disposition of the transversal fibers (**Figure 1D**).

### The Formation of the Seed Coat Throughout Olive Fruit Development

**FIGURE 2:**
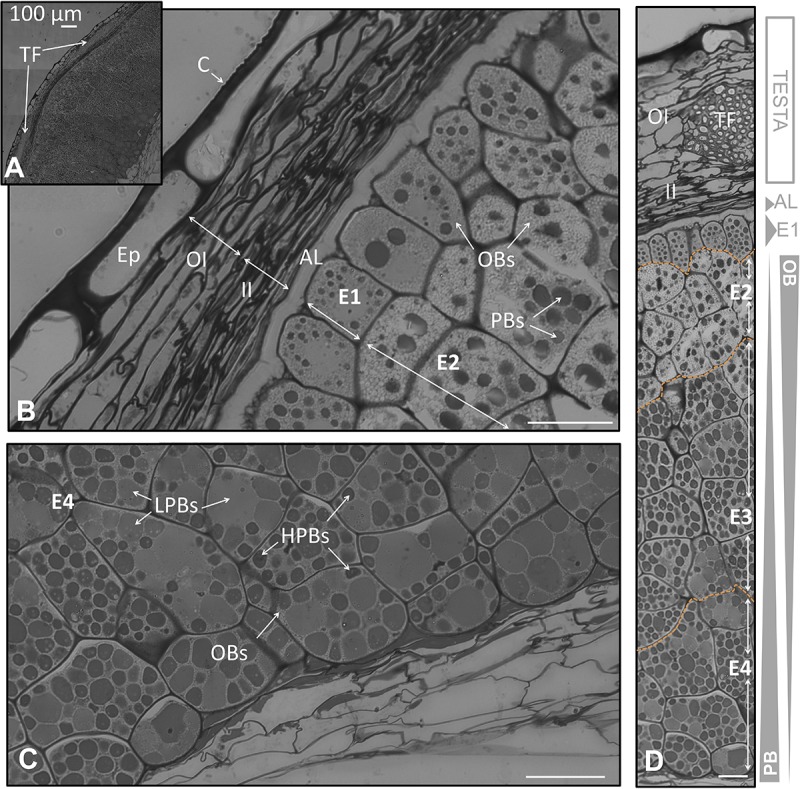
Anatomical structure of the coats and the endosperm of the olive seed 105 DAA, corresponding to green fruit. **(A)** Cross section of the testa and the endosperm. **(B)** Testa and adjacent cells corresponding to the endosperm. **(C)** Cell of the endosperm corresponding to the part in contact with the embryo. **(D)** Testa and endosperm cross section showing the differential content in lipids and proteins along it. Orange dotted lines demarcate the different layers of the endosperm. OBs, oil bodies; PBs, protein bodies; C, cuticle; Ep, epidermis; OI, outer integument; II, inner integument; TF, transversal fibers; AL, aleurone layer; E1, endosperm layer 1; E2, endosperm layer 2; E3, endosperm layer 3; E4, endosperm layer 4.

**FIGURE 3:**
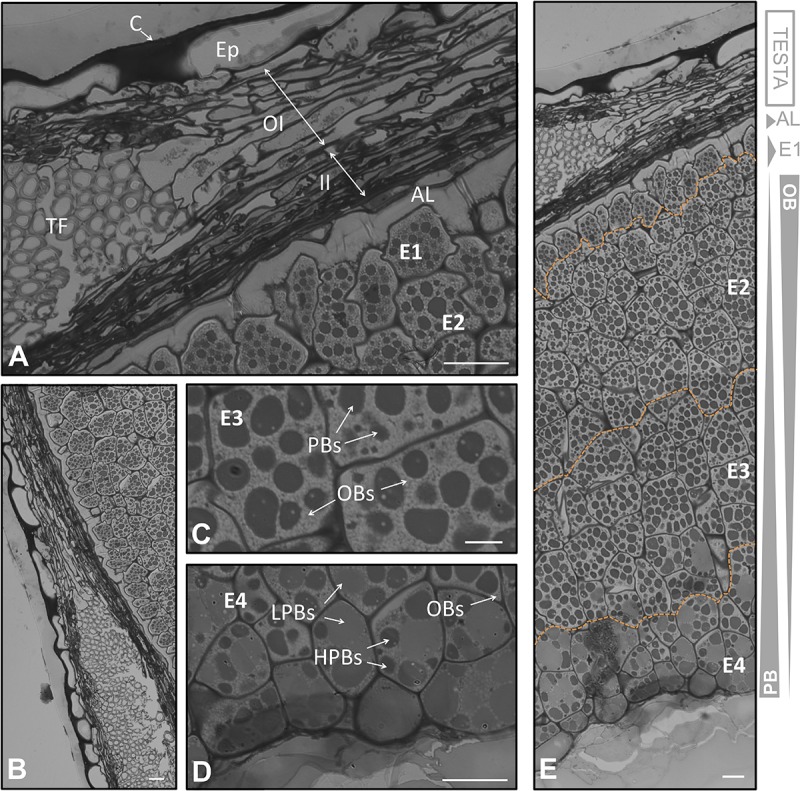
Anatomical structure of the coats and the endosperm of the olive seed 130 DAA, which corresponds to the fruit at the veraison stage. **(A,B)** Cross section of the testa and cells adjacent to the endosperm. **(C)** Cells in the middle part of the endosperm. **(D)** Cells corresponding to the part in contact to the embryo. **(E)** Testa and endosperm showing differential protein/lipid content. Orange dotted lines demarcate the different layers of the endosperm. OBs, oil bodies; PBs, protein bodies; C, cuticle; Ep, epidermis; OI, outer integument; II, inner integument; TF, transversal fibers; AL, aleurone layer; E1, endosperm layer 1; E2, endosperm layer 2; E3, endosperm layer 3; E4, endosperm layer 4.

**FIGURE 4:**
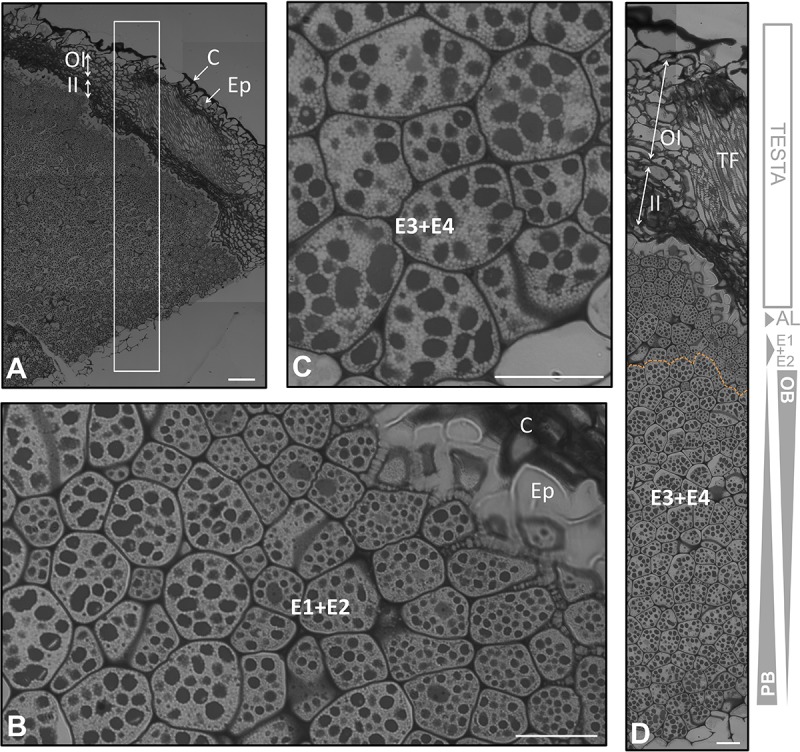
Anatomical structure of the coats and the endosperm of the olive seed 210 DAA, obtained from mature fruits. **(A)** Cross section of the testa and endosperm. **(B,C)** Cell of the endosperm close to the testa, **(C)** cells in the middle part of the endosperm. **(D)** Testa and endosperm cross section. Orange dotted lines demarcate the different layers of the endosperm. OBs, oil bodies; PBs, protein bodies; C, cuticle; Ep, epidermis; OI, outer integument; II, inner integument; TF, transversal fibers; AL, aleurone layer; E1, endosperm layer 1; E2, endosperm layer 2; E3, endosperm layer 3; E4, endosperm layer 4.

After fertilization, the integuments of the ovule normally develop into the seed coat or testa. The histological analysis of this tissue along three stages of the seed development has revealed that three layers can be distinguished: (i) mucilage or cuticle, (ii) epidermis, (iii) integument (**Figures 2**–**4**).

At the green fruit stage (105 DAA), the cuticle appeared strongly stained, forming a layer that covered evenly the non-stained and long shaped cells from the epidermis. Underneath appeared the integument composed by 8–10 well packed cells in a longitudinal orientation. The integument was divided into two parts: the outer and the inner integument; each one formed by 4–5 layers of cells. In the inner integument the cells displayed a more-flatted form, with minor intracellular spaces in comparison to the outer integument (**Figure 2B**). A cross section of the coat showed the presence of transversal fibers. These fibers crossed the integuments at the line of separation between both integuments causing a prominence of the coat (**Figures 2A,D**). This prominence causes the typical ornamentation of the olive seed that can be macroscopically distinguished.

At the veraison stage (130 DAA), a conspicuous loss of thickness of the cuticle in certain areas was detected. The cells from the epidermis appeared slightly distorted when compared to those at the green fruit stage. Besides, the start of a laxation in the cells from the outer integuments was noticed, whilst in the inner integument the cells appeared more densely packed. The transversal cells crossing the integument were observed to suffer also a light loosening, which also contributed to a progressive loss of compaction of the seed coat (**Figures 3A,B**).

At fruit maturity (210 DAA), the seed coat was characterized by the structure disorganization of the different layers. The cuticle was irregularly disposed over the epidermal cells, with a significant loss of width in some areas. The epidermis cells appeared with a patent loss of the structured disposition described for the previous stages. The same phenomena occurred in the outer, the inner integument, and the transversal fibers (**Figures 4A,B,D**).

### The Formation of the Endosperm Throughout Olive Fruit Development

The outermost layer of the endosperm (termed aleurone) was observed to be composed by longitudinal shaped cells that laid over the cells of the endosperm with a high content in lipids. At the green fruit stage (105 DAA), this layer was well developed (**Figures 2A,D**). At the veraison stage (130 DAA), no significant changes were observed in the aleurone layer, with the exception of minor modifications in the shape. The cells set off slight penetrations in the vicinity of the endosperm cells (**Figures 3A,E**). At the mature fruit stage (210 DAA), the aleurone layer seemed with a less-structured disposition compared to the previous stages. The lipid-rich cells forming the upper part of the endosperm appeared interweaved with those from the aleurone (**Figures 4A,D**).

In the olive endosperm two main types of reserve material were detected along the seed/fruit development: lipids and proteins. These substances have been already described during the olive seed formation and in the olive seedling, where they have been related in unspecified areas of the endosperm and embryo. The proteins have been reported to accumulate forming PBs, surrounded by lipids that form oil bodies (OBs) (Jiménez-López and Hernández-Soriano, 2013; Jiménez-López et al., 2015).

In the present study we have observed that the endosperm was composed by isodiametric cells with uneven distribution of PBs/OBs (**Figures 1E**, **2D**, **3D**). The gradient of PBs/OBs accumulation followed a similar pattern during the three stages considered (from 105 to 210 DAA). The cells enriched in OBs were present predominantly near the testa, with a gradual decrease of lipids in the area near the embryo. The opposite tendency was observed in the PBs. It was detected the presence of differentially stained PBs within the endosperm cells. Thus, even when considering one single cell, differential types of PB staining was noticed. Cytokinesis phenomena occurred along the tree stages of development as phragmoplasts were detected.

Attending to the disposition and the PBs/OBs content within the endosperm cells, a classification of this tissue into four layers was performed. The first layer (adjacent to the aleurone) was named endosperm 1 (E1). It was detected as a monolayer of isodiametric cells with an arranged disposition. These cells contained small PBs surrounded by small OBs (**Figures 1C,E**). Following the E1, the cells were bigger and with an untidy disposition. This area was named as endosperm 2 (E2) and was the most lipid-enriched layer (**Figures 1C,E**). The area named as endosperm 4 (E4) was highly enriched in PBs, with an increment in their size. The area named as endosperm 3 (E3) was considered as a transition between E2 and E4 as regard to the size and quantity of PBs/OBs.

Noticeable modifications in the pattern of accumulation of reserve substances were observed in the endosperm 130 DAA, corresponding to the veraison stage. The E1 layer was not so clearly differentiated from the E2 as it was in the green fruit stage. The E1 cells lost their arrangement and contained larger PBs (**Figures 3A,E**). The differences between E1, E2, and E3 were not so apparent (**Figure 2D**). However, the transition between the E3 and E4 layers was still perceptible (**Figures 3C,D**).

At the mature stage, the main characteristic of the endosperm was an increment in the homogeny of the cellular size and PB/OBs composition. The aleurone and the E1 layers were interweaved. A conspicuous differentiation could be observed, with the E1+E2 representing a single layer and E3+E4 another one (**Figures 4A,C,D**). The distribution of the storage material was similar to that described by other authors at the same stage of development (Jiménez-López and Hernández-Soriano, 2013).

### The Formation of the Cotyledon Throughout Olive Fruit Development

As described for the endosperm, the olive embryo also stocks two main kinds of storage material: lipids and proteins (Jiménez-López and Hernández-Soriano, 2013; Jiménez-López et al., 2015) that build up OBs and PBs, respectively. A deep scrutiny on the embryo histology showed an uneven distribution of this storage material mainly in the cotyledon and the radicle.

**FIGURE 5:**
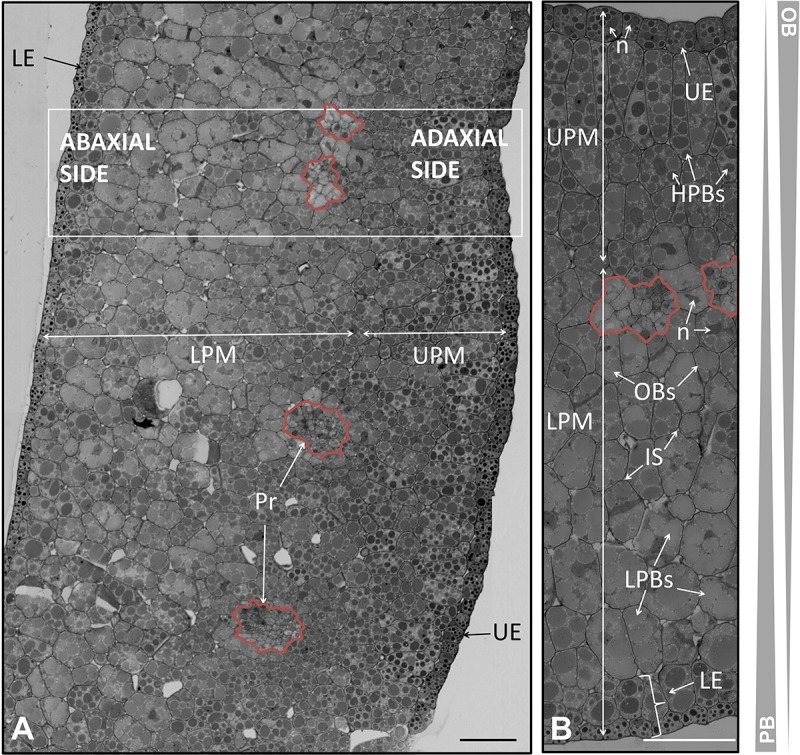
Anatomical structure of the cotyledon of the olive seed 105 DAA. **(A)** Low magnification picture of the cotyledon. **(B)** Large magnification of the cotyledon revealing the precursors of the vascular system (pointed out with a red line). OBs, oil bodies; PBs, protein bodies; UE, upper epidermis; LE, lower epidermis; LPM, lower premesophyll cells; UPM, upper premesophyll cells; Pr, procambium; IS, intracellular spaces; n, nucleus.

Observation of cross sections of the embryo at the cotyledon level (lines named “2” at **Figure 1**) 105 DAA showed the presence of a storing premesophyll tissue that appeared to consist of two zones with cells differing in shape, OBs/PBs distribution and intracellular spaces. Taking into account the orientation of the cotyledon sections, the two areas were identified as the future abaxial/adaxial sites of the leaf. In both zones, the premesophyll was covered by one/two layer of cells corresponding to the upper and lower pro-epidermis, respectively (**Figure 5**).

The pro-epidermal cells of the adaxial side, called upper pro-epidermis were cubic in shape and possessed small and strongly stained PBs surrounded by OBs. On the other hand, the under pro-epidermis was a monolayer of long shaped cells with transversal disposition. Underneath, it was noticed the presence of three layers of isodiametric cells forming the upper premesophyll (UPM). The UPM had densely packed cells with larger PBs compared to the upper pro-epidermal cells. The PBs were also surrounded by OBs. The lower premesophyll (LPM) cells occupied approximately two third parts of the cotyledon section, and their PBs were larger than those from the UPM cells. In the abaxial side it was noticed the presence of intracellular spaces. Interestingly, the PBs showed different stain intensity in both parts of the premesophyll. The lower pro-epidermis was composed by two layers of cells with different characteristics. The outermost layer possessed cubic cells with small and intensely stained PBs. Next, a layer of cells with half-way characteristics of the outermost epidermal cells and the LPM cells was observed. This layer was considered a transition as regard the cell size, PBs size, PBs stain intensity, and cell shape. The presence of nucleus was detected in all the cells along the cotyledon section (**Figure 5B**).

In between the UPM and the LPM cells, the presence of clusters of cells with irregular shape and size were distinguished, corresponding to the precambium. These cells appeared as densely packed, with nucleus, and without storage material within them (**Figure 5**).

**FIGURE 6:**
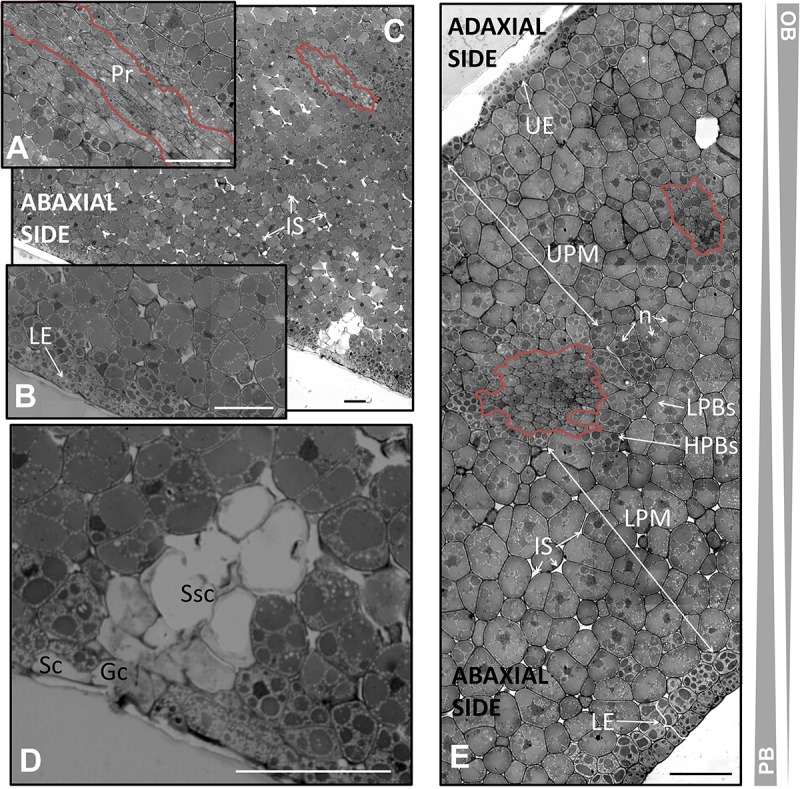
Anatomical structure of the cotyledon of the olive seed 130 DAA. **(A)** Low magnification of the cotyledon. **(B,C)** Large magnification of the cotyledon at the procambium (pointed out with a red line) and lower pro-epidermal cells, respectively. **(D)** Stoma. **(E)** Cotyledon section showing the abaxial and adaxial sites and the cells encompassing them. OBs, oil bodies; PBs, protein bodies; HPBs, highly stained protein bodies; LPBs, low stained protein bodies; UE, upper epidermis; LE, lower epidermis; LPM, lower premesophyll cells; UPM, upper premesophyll cells; Pr, procambium; IS, intracellular space; n, nucleus; Gc, guard cells; Sc, subsidiary cells; Ssc, substomatal cavity.

The analysis of the histology of the olive seed cotyledon at the veraison stage of the fruit revealed changes in the premesophyll, precambium, and epidermal cells, which were characterized by changes in the disorganization of the storage material. The nucleus was observed in the cells of all the tissues. At this stage, the presence of structures considered precursors of stoma was detected (**Figure 6**).

The upper pro-epidermis was formed at 130 DAA by a single layer of cubic cells with parallel disposition and with small and intensely stained PBs surrounded by OBs. The layer of cells under the upper pro-epidermis had suffered transversal divisions giving rise to isodiametric cells similar to those of the rest of the UPM below. The cells from the adaxial side appeared densely packed with a tendency toward homogeneity in the cell size, PBs size, and PBs staining intensity. Concerning the LPM cells, the presence of subtle changes in the size and PBs/OBs disposition was detected, leading to a homogenization of the internal organization from the UPM and LPM. The two zones were not so clearly differentiated as in the previous stage with the exception of the presence of the precambium. Moreover, in the UPM cells there was noticed a combination of low- and highly stained PBs within the same cell, being predominant the latest ones. This phenomenon also was evident in the LPM cells, where the low-stained PBs were the most abundant ones in this case. It was observed that the LPM possessed several distinctive attributes: the intracellular spaces, cells slightly bigger than those from the UPM, and PBs occupying most of the volume of the cell.

The cells forming the precambium were detected in the center of the cotyledon section. Noticeable changes in the total area of the UPM and LPM were detected in comparison with the previous stage, with an increment in the UPM and a drop in the LPM area, respectively.

Modifications in the lower pro-epidermis at the veraison stage were detected. Two layers were distinguished, both of them composed by cubic, parallelly arranged cells, and with small PBs intensely stained within them. However, the sizes of the PBs were smaller in the outermost layer than in the internal one. In both cases, PBs size, stain intensity and OBs quantity were clearly different from the cells from LPM (**Figure 6**).

Precursors of stomata were observed at the abaxial side. The lower epidermis was interrupted by the guard and the subsidiary cells. Below the stoma, a mass of non-stained cells with intracellular spaces was identified. The structure was similar to that described in *Zea mays* (Mauseth, 1988; **Figure 6D**).

**FIGURE 7:**
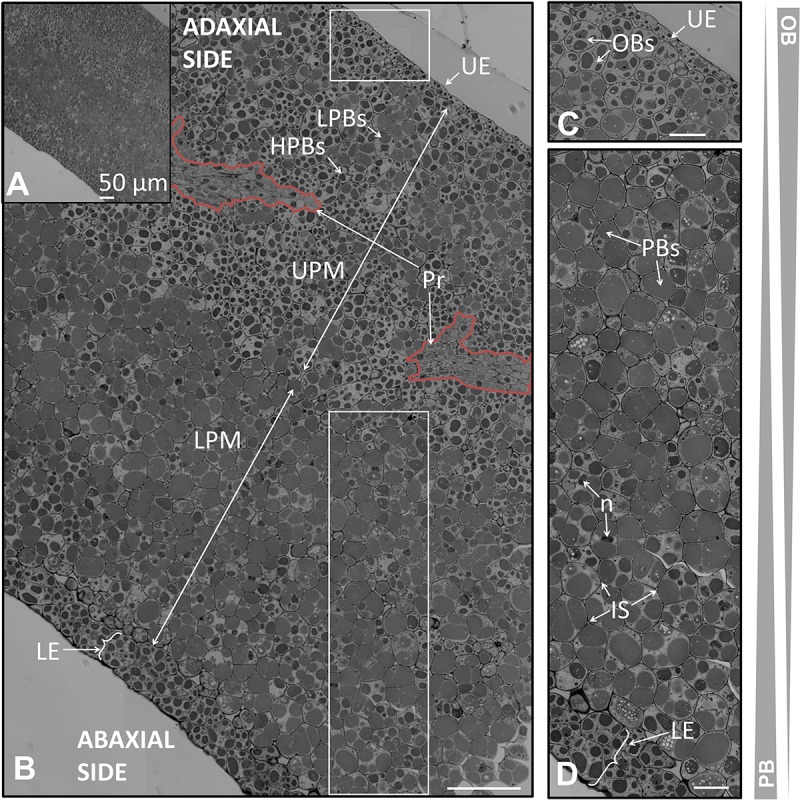
Anatomical structure of the cotyledon of the olive seed 120 DAA. **(A)** Low magnification of the cotyledon. **(B)** Large magnification of the cotyledon. **(C)** Detail of the upper pro-epidermis and UPM cells. **(D)** Detail of the lower pro-epidermis and the LPM cells. OBs, oil bodies; PBs, protein bodies; HPBs, highly stained protein bodies; LPBs, low stained protein bodies; UE, upper epidermis; LE, lower epidermis; LPM, lower premesophyll cells; UPM, upper premesophyll cells; Pr, precambium; IS, intracellular space; n, nucleus.

The study of the anatomy of the olive cotyledon in the mature seed corresponding to 210 DAA showed a defined structure, with clear precursors of the spongy and palisade mesophyll. The imbalanced distribution of the storage material within the cells of the mesophyll was observed to be the main characteristic of this stage (**Figure 7**).

The upper pro-epidermis contained a monolayer of flattened cells disposed parallel in the plane to the surface. These cells were observed to be highly enriched in OBs and small PBs (**Figure 7C**).

The UPM cells occupied half of the cross section of the cotyledon and they were filled by numerous OBs surrounding the PBs. There was a mixture of highly stained and low stained PBs within the cells, mainly dominated by the highly stained ones. The cells from the LPM had a lower OBs content that surrounds the large PBs. Poles apart, the low stained PBs were dominant over the high stained ones within the cells of the LPM (**Figures 6B,D**).

The precambium appeared among the UPM as a group of long shaped cells without storage material within them (**Figure 7B**). At the mature stage the procambium did not show mature xylem or phloem elements. The lower epidermis was detected to be composed by two layers of cells with a non-arranged disposition, with highly stained PBs and elevated quantities of OBs (**Figure 6D**). The nucleus was observed in all the layers of cells across the cotyledon section.

### The Formation of the Radicle Throughout Olive Fruit Development

**FIGURE 8:**
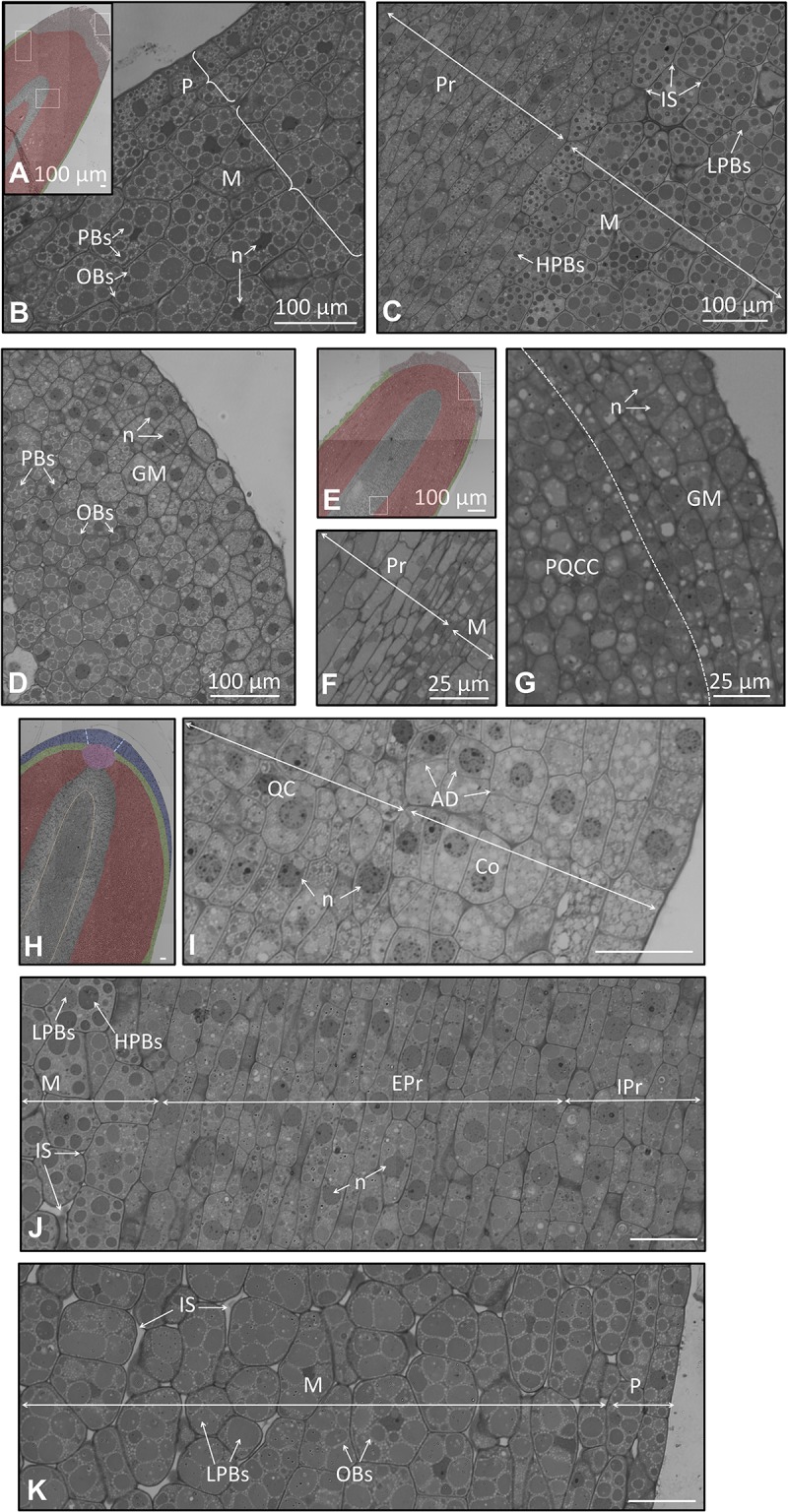
**(A–G)** Longitudinal section of the embryo at the radicle part in a seed 105 and 130 DAA. **(A)** Low magnification radicle showing the different kind of cells 105 DAA. In light red appears the grown meristem at the apex; in green the protoderm; in gray the procambium. **(B)** Large magnification of the radicle showing a detail of the protoderm and the meristem 105 DAA. **(C)** Large magnification of the cell of the procambium and the meristem 105 DAA. **(D)** Large magnification of the ground meristem at the very apex 105 DAA. **(E)** Low magnification radicle showing the different kind of cells 130 DAA. In light red appear the grown meristem at the apex; in green the protoderm; in gray the procambium. **(F)** Cell of the procambium and the meristem 130 DAA. **(G)** Cell of the procambium and the meristem 130 DAA. **(H–K)** Longitudinal section of the embryo at the radicle in a seed 210 DAA. **(H)** Low magnification radicle section showing the different types of cells. In red appears the meristem; in green the protoderm; in blue the root apex, in gray the procambium. Orange dotted line demarcates two different types of cells within the procambium; blue dotted line demarcates the columella. **(I)** Detail of the radicle at the root apex and quiescent center. **(J)** Large magnification picture of the meristem and the procambium. **(K)** Cells of the protoderm and the meristem. P, protoderm; M, meristem; Pr, procambium; QC, Quiescent Centre; C, columella.

Sections of the radicle taken form seeds at the green fruit stage showed the presence of three different kind of cells corresponding to the protoderm, ground meristem, and procambium, respectively (**Figure 8A**). The cells from the apex, which form to the ground meristem had a high degree of compaction and possessed large nuclei. The PBs were also large being surrounded by small OBs. The apical ground meristem cells suffered anticlinal divisions (**Figure 8D**). Regarding the protoderm, two layers of long shaped cells were observed, being the PBs small and intensely stained (**Figure 8B**). Underneath, a gradual change in the cell shape and the characteristics of the stored material within the cells was detected, giving rise to isodiametric cells with low-stained and large PBs. These cells were bigger than those forming the protoderm and the presence of intracellular spaces among them was detected. The procambium cells were long-shaped, devoid of storage material, and lacking intracellular spaces among them, which allowed differentiating them from the meristem cells. In between the procambium and the meristem, cells appeared as a transition concerning to the shape and the PBs/OBs content (**Figure 8C**).

At the veraison stage, few changes in the histology of the radicle were observed (**Figures 8E–G**). These changes corresponded mainly to the meristem, which appeared less packed. The cells at the apex of the meristem were an exception, with a high degree of compaction among the cubic-shaped cells.

At the mature fruit stage (210 DAA), we observed the presence of notorious changes in the organization and differentiation of the cells of the radicle (**Figures 8H–K**). The procambium appeared as a central bundle in the midpoint of the radicle. At the distal end of the procambium, the quiescent center was visible, mainly characterized by the disposition of the cells around a central point. In the above part, the cells of the columella and root apex displayed an arranged organization (**Figure 8I**). On the left and right sides of the quiescent center it was noticed that the cells suffered a progressive change in the shape and content of storage material to finally give rise to the meristematic cells. Below the quiescent center, the procambium comprises two areas. The area located in the middle was composed by isodiametric cells containing low-stained PBs. The external area was comprised of long-shaped cells without OBs, nor PBs. In both cases, the presence of the nucleus within the cells was perceptible, as well as the absence of storage material (**Figure 8J**). The protoderm was identified as two layers of long-shaped cells longitudinally arranged. These cells were differentiated from the adjacent meristematic cells since the latter possessed intracellular spaces, large cellular size, large PBs, and non-well-structured cells (**Figure 8K**).

**FIGURE 9:**
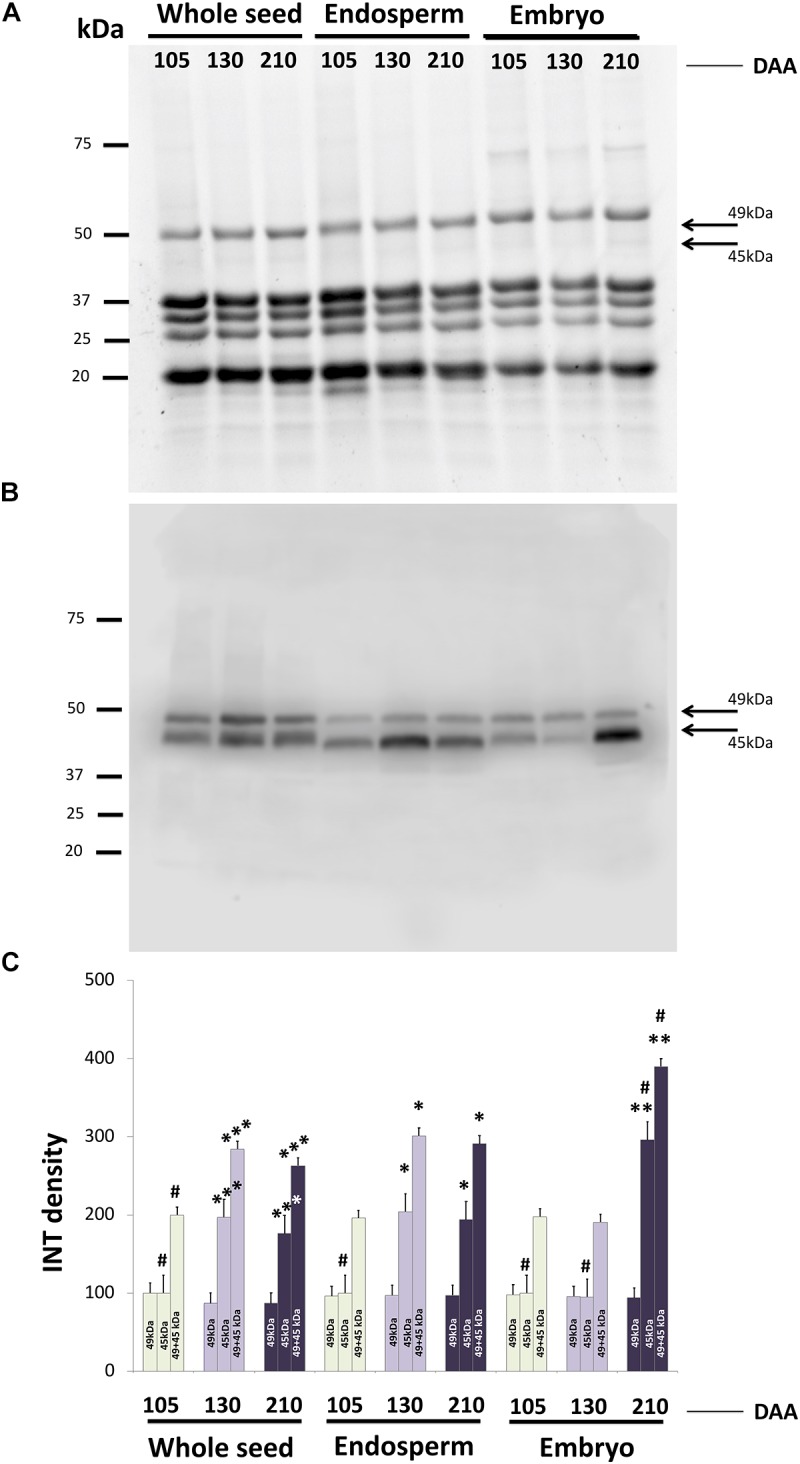
SDS-PAGE profiles of and Western blotting analysis of β-conglutins in samples of whole seeds, isolated endosperm plus testa and embryo from olive fruits at different developmental stages. **(A)** SDS-PAGE profiles showing conspicuous bands corresponding to 11S peptides under denaturing, reducing running conditions. **(B)** Western blotting after using the anti β-conglutin antibody. **(C)** Densitometry of the reactive bands to the antibody. Green bars: green fruit (105 DAA). Light purple bars: veraison fruit (130 DAA). Dark purple bars: mature fruits (120 DAA). ^∗^*p* < 0.001 versus the corresponding endosperm sample at 105 DAA; ^∗∗^*p* < 0.001 versus the corresponding embryo sample at 105 DAA; ^∗∗∗^*p* < 0.001 versus the corresponding whole seed sample at 105 DAA; ^#^*p* < 0.001 among all samples of the same band category (49, 45, or 49+45 kDa).

SDS-PAGE protein profiles of whole seeds, isolated endosperm (+testa) and embryo at different DAA were resolved by SDS-PAGE under reducing conditions, as displayed in **Figure 9A**. Conspicuous bands of proteins appear corresponding to the peptides p1 to p5 as described by Alché et al. (2006), which represent different peptides integrating the highly abundant 11S SSPs. The Western blotting profile after probing with the anti-β-conglutin primary antibody showed two reactive bands of c.a. 45 and 49 kDa, respectively present in all extracts, although with different relative intensities (**Figure 9B**). Relative quantification of each one of the reactive bands in all samples showed bands of 49 kDa evenly distributed in the endosperm and embryo tissues, with little changes in their intensity through the time developmental course. Contrary, the bands of 45 kDa presented noticeable changes in their intensity, particularly along the developmental stages for a given tissue (endosperm and embryo). The added intensities of both bands for each stage exhibited an increasing trend in the overall amount of β-conglutin along endosperm, embryo, and whole seed development (**Figure 9C**). Relative amount of β-conglutins was higher in the embryo compared to the endosperm.

**FIGURE 10:**
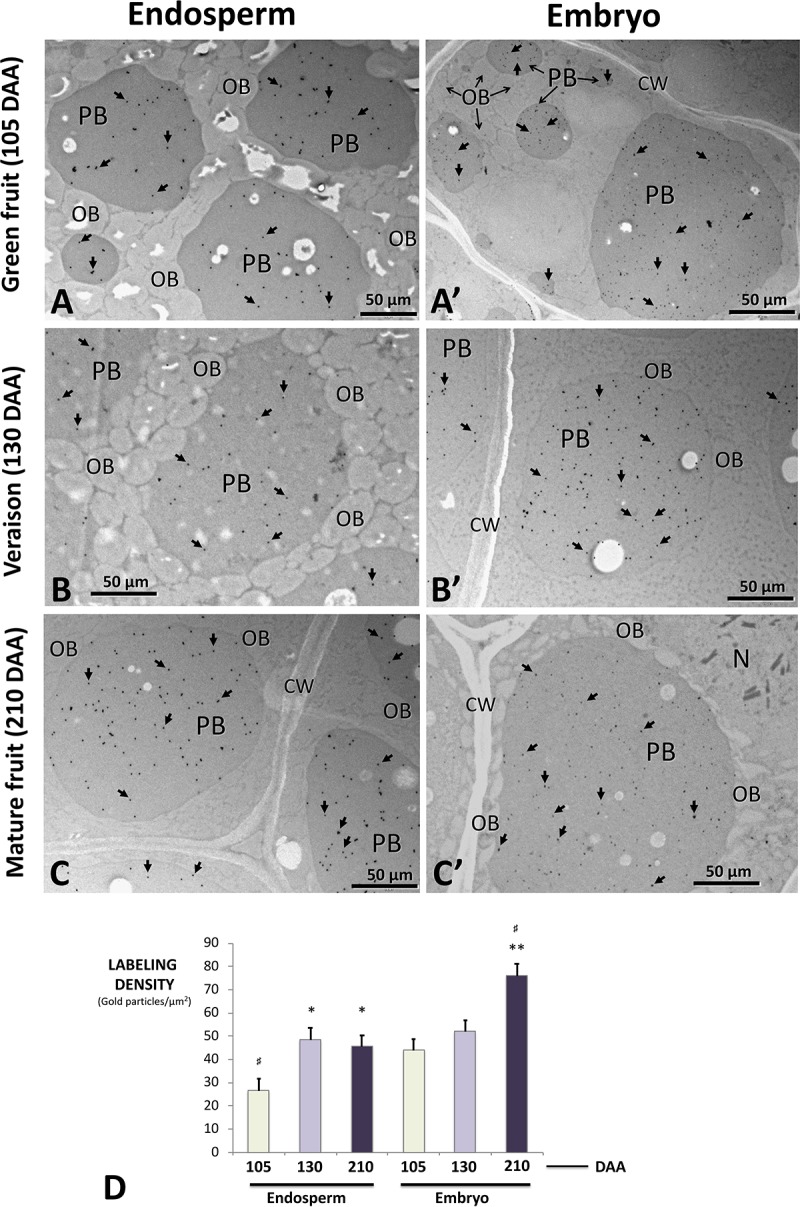
Transmission electron microscope (TEM) immunolocalization of β-conglutins in samples of endosperm plus testa **(A–C)** and embryo **(A′–C′)** from olive fruits at different developmental stages. Gold particles (arrows) are specifically decorating protein bodies of different sizes. Lipid bodies, cell wall, and the nucleus are devoid of gold particles. **(D)** Quantification of labeling density in the protein bodies of both tissues at the different stages analyzed. Green bars: green fruit (105 DAA). Light purple bars: veraison fruit (130 DAA). Dark purple bars: mature fruits (210 DAA). CW, cell wall; OBs, oil bodies; N, nucleus; PBs, protein bodies. Magnification bars: 50 μm. ^∗^*p* < 0.001 versus the corresponding endosperm sample at 105 DAA; ^∗∗^*p* < 0.001 versus the corresponding embryo sample at 105 DAA; ^#^*p* < 0.001 among all samples.

Immunolocalization studies using the anti-β-conglutin primary antibody yielded an intense labeling by gold particles specifically located in the PBs present in the endosperm and the embryo all-through the seed developmental stages (**Figures 10A–C,A’–C’**). Labeling in the lipid bodies, any other cell structures (cell wall, nucleus, and testa) and in the negative controls processed by either omitting the primary antibody or using the pre-immune serum (not shown) was negligible. A statistically significant and progressive increase of labeling density in the PBs present in both the endosperm and the embryo was observed (**Figure 10D**). The overall density of labeling was significantly higher in the embryo than in the endosperm (**Figure 10D**).

### Seed Weight Variability Among Olive Cultivars

The weight of the whole fruit was a variable parameter in the cultivars submitted to the present study (**Supplementary Figures S1A,B**). They ranged from an average of 1.28 g in ‘Lechin’ to 4.91 g in ‘Ombliguillo.’ Similarly, the average weights of the mesocarps were comprised between 0.89 g in ‘Lechín’ to 4.00 g in ‘Ombliguillo.’ Besides, the data obtained from the average weight of the endocarp oscillated from 0.39 g in ‘Lechín’ to 0.99 g in ‘Gordal de Alhama.’ The weights of the whole fruit and that of the pulp (mesocarp + epicarp) showed to have a positive correlation (**Supplementary Figure S1C**). As regard to the number of seeds found within each endocarp, six of the cultivars showed just one seed, whereas in the other six cultivars we managed to observe the presence of two seeds per endocarp in some of the fruits.

Focusing on the seed tissues we identified that the average weights of the complete seeds measured in the 12 cultivars ranged from 0.040 in ‘Lechín’ to 0.101 g in ‘Azul.’ The testa ranged from to 0.005 g in the cultivar ‘3’ to 0.030 g in the cultivar ‘Hojiblanca.’ The endosperm ranged from 0.024 g in ‘Picual’ to 0.059 g in ‘Loaime.’ The embryo weights were comprised among 0.011 g in ‘Picual’ to 0.032 g in ‘Nevadillo’ (**Supplementary Figure S1D**).

As regard to the olive yield, measured as the ratio complete fruit/pulp weight, the obtained data showed the lowest ratio for ‘Llorón’ (1.20) while the highest one corresponded to ‘Picual’ with a ratio of 1.47. On the other hand, the fruit/endocarp ratio oscillated between 3.15 in ‘Picual’ to 5.77 in ‘Llorón’ (**Supplementary Figure S1E**). The endocarp/seed ratio showed values among 4.83 in ‘Azul’ to 14.72 in ‘Picual.’ The seed/embryo ratios oscillated between 2.15 in ‘Nevadillo’ to 4.58 in ‘Ombliguillo.’ In the case of the seeds containing two seeds per endocarp, each seed was weighted as independent sample. Spearman correlation between the weights of endosperm/embryo, seed/testa, seed/endosperm, and seed/cotyledon for different olive cultivars, as well as the registered presence of some fruits of the cultivar containing more than 1 seed is displayed in **Supplementary Figure S1F**.

## Discussion

Endocarp morphology is a widely accepted pomological signature for olive tree identification and classification of cultivars based on the presence of morphological differences (Barranco and Rallo, 1984; Rallo et al., 2005), which has been later evidenced to be in accordance with results obtained by molecular methods like simple sequence repeat (SSR) screening (Fendri et al., 2010). Within the endocarp, the olive seed represents a potential source of nutrients and biological elements of high interest, in addition of representing an additional varietal mark as demonstrated in the present work. Such designed potential will allow increasing the added value of this material, which is frequently disposed of concomitantly with olive processing residues. The olive seed can also be used as a source of genetic variability of interest for the development of breeding programs, in combination with the vegetative propagation of the resulting individual of interest (Morales-Sillero et al., 2012).

Histological structure of the olive seed doesn’t substantially differ from those of dicots as described here; however, the distribution of the different tissues and their development has to be assessed in order to gain knowledge and establish parameters of maturity, which make easier the analysis of the expression and the presence of the compounds of interest, as is the case of 11S proteins (Alché et al., 2006; Jiménez-López et al., 2015) and the present case of 7S proteins described here. Such studies may help to define further technological developments, i.e., for sub-fractioning olive seed in order to enrich certain components, which could be majority present in a particular fraction. Also, these analyses may help to identify histological parameters relevant for seed and fruit physiology. Thus, the seed coats from different species have been analyzed for a variety of purposes such as the generation of a dichotomous key (Kaplan et al., 2009), or to analyze implications in key physiological roles like viability, dormancy and early control of germination (De Giorgi et al., 2015). The seed coat from the olive tree contains a well-defined cuticle covering the epidermis, which could be involved in key physiological roles. The intense staining might indicate a major presence of proteins, analogously as described in the grape seed coat, which is also rich in polysaccharides (Cadot et al., 2006). Proteome analysis in *Arabidopsis* has revealed the presence of proteins unique to mucilage responsible of alterations of its structure and mechanical alteration of the primary cell wall (Arsovski et al., 2010; Haughn and Western, 2012; Tsai et al., 2016). The key role of the seed cutin has also been associated to soil erosion (Engelbrecht et al., 2014). However, the protein complexity of the seed coat in the olive seed is still unrevealed.

We observed a general laxation and disarrangement of the coats that could be involved in the need to have access to oxygen needed in the germination process. The three-dimensional study of the *Arabidopsis* seed revealed a putative network of intercellular air space that allows gas exchange for germination (Cloetens et al., 2006). The X-ray in-line phase tomography performed in maize is as practical tool for the detection of other characteristic non-detectable by conventional microcopy methods, like the metabolic state and the water content (Rousseau et al., 2015). The olive seed presents intracellular spaces and discontinuous in their structures putative involved in the need for the gas exchange, water intake or metabolic activity mainly in the mature stage and prior to the germination process. The presence of a well-defined aleurone layer in the olive seed has been described in the present study. This structure changes form a well-structured disposition in the green stage to disorganization in the mature stage that could be involved in some way in the easy removal of the seed coat at the mature stage.

As regards to the endosperm, Floyd and Friedman (2000) provided the first insight into how different endosperm developmental patterns are evolutionarily and developmentally related. The study of the endosperm possesses an increasing interest further than the long-established role of the endosperm as nourishment and mechanical barrier. The endosperm is capable of sensing environmental signals and interacts with the embryo establishing a bidirectional communication (Yan et al., 2014). The endosperm in the olive tree showed a clear change as regard to the organization and quantity of the OBs/PBs. These data were similar to those of the embryo. Moreover, it was found the presence of differentially stained PBs in both tissues. Thus, the composition of both, the proteins and the lipids could be differentially accumulated. The analysis of the fatty acid composition of the endosperm and embryo was detected to be different in *Arabidopsis* probably due to an hormonal regulation (Penfield et al., 2004) and later confirmed in both, *Arabidopsis* and *B. napus* (Li et al., 2006). These authors also observed that the fatty acid profile was different among embryo tissues. Finally, the apical meristems consist in three types of tissues that correspond to protoderm, ground meristem and procambium. The procambium is differentiated early in the development (60 DAA), however, the proper phloem and xylem did not appear differentiated in the mature seed. These events correlates to those previously described by Zienkiewicz et al. (2011) that pointed out the complete cellular organization of the leaf olive mesophyll is achieved 16 days after germination.

The study of the olive embryo and endosperm reveals the 11S protein as the most abundant one in these tissues (Alché et al., 2006). However, no studies about the presence of other specific proteins are available till the moment, whereas the present study confirms that 7S-type SSPs (β-conglutins) are also relevant constituents of both the endosperm and the embryo. Both proteomic and transcriptomic analysis aimed to agronomical improvements have shown for example the seed coat to function as a specialized secondary cell wall (Haughn and Western, 2012), to be involved in endosperm permeability, seed viability, and seed dormancy which correlates with higher levels of seed lipid oxidative stress (De Giorgi et al., 2015), with implication in specific functions that affects the seed composition, seed permeability, and hormonal regulation (Verdier et al., 2013). The study of both the proteome and the transcriptome of the olive seed (currently being approached) could represent interesting tools for multiple purposes, including the study of specific proteins involved in organoleptic properties of the olive oil. Thus, the presence of seed enzymes involved in the lipoxygenase pathway, enzymatic activities metabolizing 13-hydroperoxides other than hydroperoxide lyase, alcohol dehydrogenase, and alcohol acyltransferase activities among others would provide multiple esters in the olive oil (Luaces et al., 2003, 2007).

Within the increasing demand for plant-derived proteins as components of functional foods in the nutraceutical industry and as an alternative to expensive and less-environmental-friendly production of animal protein, β-conglutins are considered an economical dietary source of good quality protein. Also, they have positive effects on many human health dysfunctions, as many of the seeds containing β-conglutins are protein- and fiber-rich, low in fat and starch, and have a very low glycemic index (Arnoldi, 2008; Duranti et al., 2008). Positive effects have been described for these proteins on blood pressure, risk of cardiovascular disease and the prevention and treatment of type 2 diabetes, by modulating the insulin signaling pathway and diminishing inflammation (Lima-Cabello et al., 2017a). For the olive seed, preliminary work (unpublished) indicates the presence of anti-inflammatory components in the flours derived from this material. However, the direct involvement of β-conglutins in these effects is yet to be analyzed.

Expression of β-conglutins in the olive seed tissues is remarkable as shown here, with at least two forms of the protein reactive to the antibody, which might indicate the presence of a protein maturation process, as it is the case of the olive 11S SSPs (Alché et al., 2006), and has been proposed for β-conglutins (Duranti et al., 2008). The 49 kDa form of the protein shows a constitutive presence in the endosperm, embryo, and whole seed, whereas the 45 kDa form displays developmental changes as well as slight tissue differences. The accumulated presence of both β-conglutin forms indicates that this protein is progressively accumulated in the seed, through the developmental process, and that the relative amount of β-conglutins was higher in the embryo compared to the endosperm. Olive seed development was already characterized as a tissue-dependent process characterized by differential rates of legumin accumulation and PB formation in the main tissues integrating the seed (Jimenez-Lopez et al., 2016) on the basis of the accumulation of 11S legumin proteins. Such developmental pattern is then shared by β-conglutins as well. The relationships between the 45 and 49 kDa forms of the protein must be stablished through future work. Proteomic and transcriptomic work in course will serve the basis for this information, and will help to determine whether these correspond to maturation forms of the protein or the result of the expression of differential genes. These studies will also determine the presence of embryo- and endosperm-specific proteins, as have been recently identified in *Phoenix dactylifera* (Sekhar and DeMason, 2017).

Lupin β-conglutins are located in the endosperm and cotyledonary PBs, as shown by immunocytochemical experiments carried out here, and as it is also the occurrence with olive 11S legumins. As showed by Duranti et al. (2008), the covalent integrity is not apparently a pre-requisite for β-conglutin to be correctly deposited in these cellular structures, since the mature β-conglutin from lupin dry seeds appeared already proteolytically cleaved in a number of sites, giving rise to complex SDS-PAGE patterns. Immunolocalization of β-conglutins in the olive PBs likely reflects the localization of both the 45 and 49 kDa forms of the protein, as they are both recognized by the antibody. Quantification of the labeling in the PBs is consistent with the quantification of the signal of the 49+45 kDa bands in Western blotting experiments, showing an incremental presence of these proteins through the maturation process, analogous to that of 11S proteins, which is concomitant with the increased presence of PBs in all the tissues analyzed here. Also, the higher presence of ß-conglutins in the embryo compared to the endosperm was verified in the immunocytochemical experiments.

Both the histological features and analytical characteristics and the localization of the olive seed β-conglutins were also determined at longer times after anthesis (240 DAA). Such parameters did not differ substantially from those displayed here for 210 DAA in the cultivar ‘Picual’ and therefore were not shown in the present work. This may suggest that maturation of the seed ends before the maturation of the pulp in the olive fruit.

The distinctive character of the olive endocarp morphology and size amongst olive cultivars, previously reported by Barranco and Rallo (1984), was also verified in the present work. However, in this case the differences were also assessed as regard to the main parameters of the different constituents of the seed. Although differences among cultivars exist, some general directions can be detected. As an example, the weights of the whole fruit and that of the mesocarp were detected to have a positive correlation for all cultivars, whereas the weight of the whole seed was positively correlated with the weights of the individual components (endosperm and cotyledon) for most cultivars, and on the contrary, no correlation was detected between the weight of the whole seed and the weight of the testa for most cultivars. Such relationships may have particular meaning for future and potential uses of particular cultivars for the extraction of seed derived components, as it is the case of polyphenols (work in progress). In addition, the endocarp is considered to represent an evolutionary strategy for seed protection and dispersal (Dardick et al., 2010). Therefore, their size, and that of the different components of the seed should be further analyzed in relation to their dispersion efficiency, viability, ability of germination and vigor for the different olive cultivars, and particularly for wild olives, which are mainly propagated by seeds. This is one of the objectives of several research projects funding the present work. Also, moderate and severe reductions in water availability proportionately decrease endocarp expansion and prolong the sclerification, delaying the date of physically perceived hardening but not affecting the final degree of endocarp sclerification (Hammami et al., 2013). Therefore, the analysis of the hardening dynamics of the endocarp and the final size of the endocarps might be used as a marker for biological studies and crop management, as well as a marker for cultivar tolerance to water availability.

## Conclusion

The described anatomy and histological distribution of the olive seed of the ‘Picual’ cultivar, allows identifying the main features typical of dicots within a developmental time frame. Cell storage structures (PBs and OBs) present a well-defined pattern of accumulation, with complementary distribution in the olive seed tissues.

Seed storage proteins of the 7S-type (β-conglutins) are relevant components of all olive seed tissues, displaying an accumulative pattern concurrent with the development of the seed and fruit. These proteins are present in at least two peptide forms, and are subcellularly associated to PBs in the different tissues analyzed.

Moreover, a panel of other olive cultivars has been used to compare the weight contribution of the different tissues to the seed, seed weight variability, and the number of seeds per fruit.

These hallmarks will help to settle the basis for future studies related to the location of different metabolites along the olive seed and mesocarp development, and therefore helping to assess the appropriate ripening stage for different commercial and industrial purposes.

## Author Contributions

AZ and JA designed the experimental structure of the work and redacted the manuscript. AZ performed the experiments, observations, image capture, and analysis of the results, whereas MM’-A performed TEM immunocytochemical detection and signal quantitation. JJ-L was particularly involved in the work with the databases and tools on the web servers for prediction of synthetic peptide and the generation of the antibody. EL was responsible for Western blotting experiments and analysis. All authors read and approved the manuscript.

## Conflict of Interest Statement

The authors declare that the research was conducted in the absence of any commercial or financial relationships that could be construed as a potential conflict of interest.

## Supplementary Material

FIGURE S1 **(A)** De-stoned seeds from 18 fruits cv. ‘Picual’ as an example. The endocarp and seeds are shown. **(B)** Dissection of the tissues from a ‘Picual’ seed. Up to down: endocarp, testa, endosperm, and embryo. **(C–E)** Representation of the different parameters in a total of 12 olive cultivars, including ‘Picual’ (arrow). Scale bars: 20 mm. **(F)** Spearman correlation of several weights of the seed tissues form different olive cultivars. (+/−) indicate positive/negative correlation (*p* < 0.01). Y/N (Yes/No) indicates whether some fruits of the cultivar contained more than 1 seed.
