# Supplementary figures and images for: Histological Features of the Olive Seed and Presence of 7S-Type Seed Storage Proteins as Hallmarks of the Olive Fruit Development
Source: Front Plant Sci. 2018 Oct 12;9:1481. doi: 10.3389/fpls.2018.01481 (PMC6194196; doi:10.3389/fpls.2018.01481)

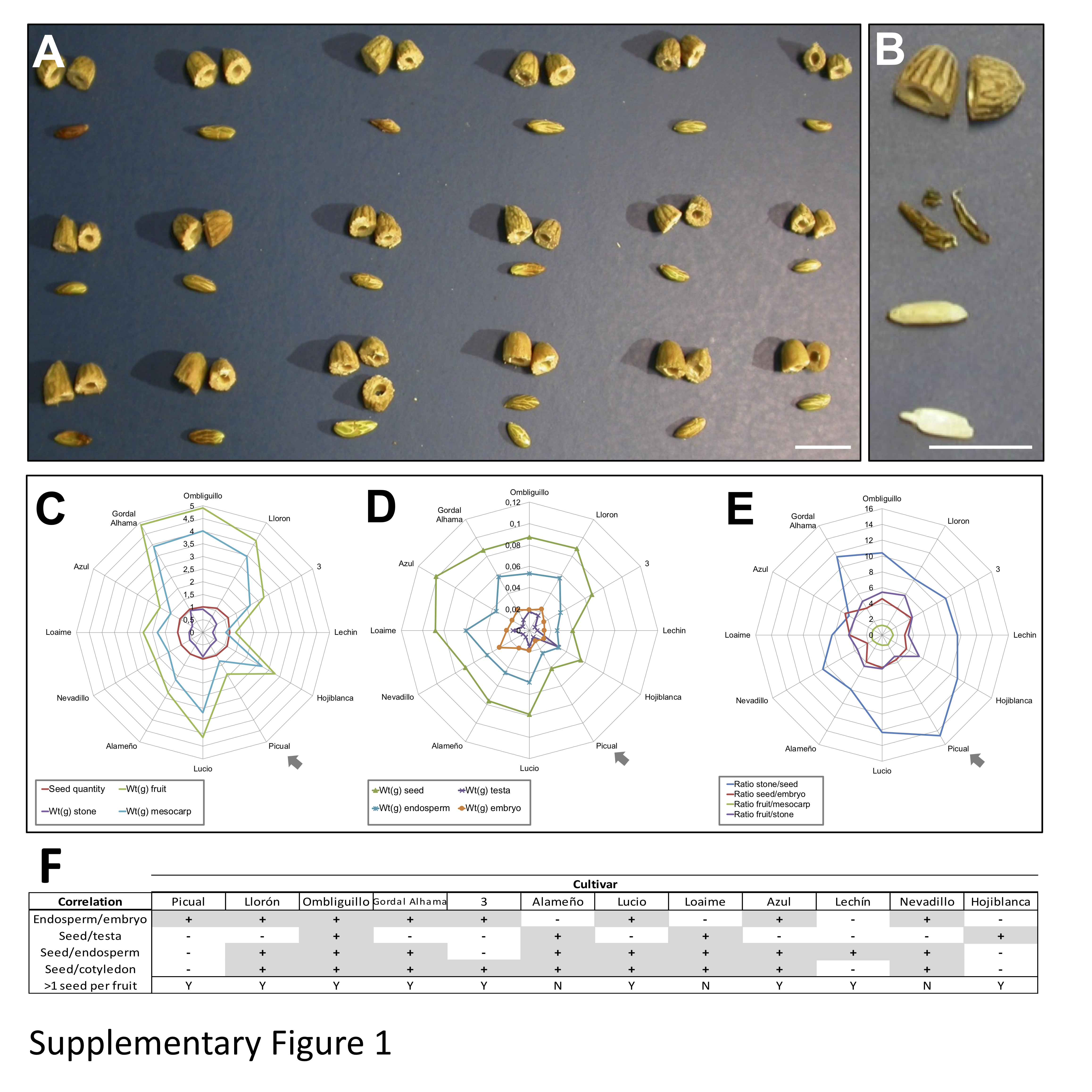

Supplement: FIGURE S1 — (A) De-stoned seeds from 18 fruits cv. ‘Picual’ as an example. The endocarp and seeds are shown. (B) Dissection of the tissues from a ‘Picual’ seed. Up to down: endocarp, testa, endosperm, and embryo. (C–E) Representation of the different parameters in a total of 12 olive cultivars, including ‘Picual’ (arrow). Scale bars: 20 mm. (F) Spearman correlation of several weights of the seed tissues form different olive cultivars. (+/−) indicate positive/negative correlation (p < 0.01). Y/N (Yes/No) indicates whether some fruits of the cultivar contained more than 1 seed. [file Image_1.JPEG]
